# Loss of Scribble Promotes Snail Translation through Translocation of HuR and Enhances Cancer Drug Resistance*

**DOI:** 10.1074/jbc.M115.693853

**Published:** 2015-11-02

**Authors:** Yi Zhou, Renxu Chang, Weiwei Ji, Na Wang, Meiyan Qi, Yi Xu, Jingyu Guo, Lixing Zhan

**Affiliations:** From the Key Laboratory of Nutrition and Metabolism, Key Laboratory of Food Safety Research, Institute for Nutritional Sciences, Shanghai Institutes for Biological Sciences, Chinese Academy of Sciences, University of Chinese Academy of Sciences, Shanghai 200031, China

**Keywords:** apoptosis, cancer biology, cancer therapy, cell polarity, drug resistance, cisplatin, HuR, Scribble, Snail

## Abstract

Drug resistance of cancer cells to various therapeutic agents and molecular targets is a major problem facing current cancer research. The tumor suppressor gene *Scribble* encodes a polarity protein that is conserved between *Drosophila* and mammals; loss of the locus disrupts cell polarity, inhibits apoptosis, and mediates cancer process. However, the role of Scribble in drug resistance remains unknown. We show here that knockdown of Scribble enhances drug resistance by permitting accumulation of Snail, which functions as a transcription factor during the epithelial-mesenchymal transition. Then, loss of Scribble activates the mRNA-binding protein human antigen R (HuR) by facilitating translocation of HuR from the nucleus to the cytoplasm. Furthermore, we demonstrate HuR can recognize AU-rich elements of the Snail-encoding mRNA, thereby regulating Snail translation. Moreover, loss of Scribble-induced HuR translocation mediates the accumulation of Snail via activation of the p38 MAPK pathway. Thus, this work clarifies the role of polarity protein Scribble, which is directly implicated in the regulation of developmental transcription factor Snail, and suggesting a mechanism for Scribble mediating cancer drug resistance.

## Introduction

Drug resistance is a common cause of cancer treatment failure, impeding the development of effective cancer therapies and limiting the efficacy of both traditional chemotherapeutic agents and more recently discovered drugs (1). At the same time, tumors are highly adaptable and able to activate alternative survival or death signaling pathways, thereby yielding drug resistance (2, 3). This multiplicity of pathways highlights the urgency to investigate the molecular and cellular mechanisms that lead to cancer cells drug resistance.

Scribble was first identified in *Drosophila melanogaster* as the product of a neoplastic tumor suppressor gene (4). The Scribble, Discs large (Dlg), and Lethal giant larvae (Lgl) proteins comprise an evolutionarily conserved polarity complex that localizes to the basolateral side of the epithelial cell membrane, where the complex regulates epithelial cell apico-basal polarity. Loss of *Scribble* disrupts apical basal polarity and junction integrity, inducing inappropriate proliferation and tissue overgrowth (5). *Scribble* has been documented to have important roles in cancer development and progression, including in colon and breast cancer (6, 7). Loss of *Scribble* results in cancerous overgrowth of imaginal discs in *Drosophila* (8). The Scribble protein has been shown to cooperate with c-Myc to induce tumors by blocking activation of an apoptotic pathway (9). Structural analysis of the Scribble protein reveals the presence of 16 leucine-rich repeats at the N terminus and 4 PDZ (PSD95, Discs large and Zonula adherens-1) domains at the C terminus. Both leucine-rich repeats and PDZ domains are protein-protein interaction modules that likely are involved in several distinct cell signaling pathways. In cell competition, Scribble knockdown (KD) cells are apically extruded from the epithelium when surrounded by normal cells, this process depends on activation of the p38 MAPK pathway (10). Separate work demonstrated that Scribble can also regulate cancer cell apoptosis through the Rac/JNK/c-Jun pathway in mammary epithelial cells (9). Moreover, inducing expression of Scribble was sufficient for tight junction formation by suppression of ERK phosphorylation (4). However, the mechanism underlying the tumor suppressor function of Scribble in cancer cell drug resistance is still unknown.

Human antigen R (HuR)^3^ is a member of the Hu protein family with homology to the *Drosophila* embryonic lethal abnormal vision (ELAV) protein. HuR has been directly implicated in cell division, cell differentiation, replicative senescence, carcinogenesis, and stress responsiveness (11). High levels of cytoplasmic HuR are associated with poor differentiation, large tumor size, and reduced survival in patients (12). HuR protein localizes predominantly in the nucleus, but has been shown to translocate to the cytoplasm in times of cellular stress, where the protein affects mRNA stability and translation. The transport of HuR across the nuclear envelope is mediated by cell cycle-dependent kinase 1 (Cdk1), protein kinase C (PKC), and p38 (11). Of note, PKC and p38 MAPK are known to phosphorylate HuR, and this interaction has been demonstrated to affect HuR cytoplasmic translocation and promote cell resistance to doxorubicin (13).

Because cell polarity normally is required to establish and maintain cell integrity and function in epithelial tissues, loss of cell polarity is an important aspect of EMT development (14, 15). Furthermore, emerging evidence suggests that EMT is regulated by a number of transcription factors, termed “EMT inducers,” including Snail, Twist, ZEB1/2, E47, and KLF8 proteins (16). Snail is a major EMT inducer; its activity affects disparate intracellular signaling pathways, ultimately converging on the EMT (17). Notably, although Snail acts primarily as a key inducer of EMT, this factor also plays an important role in cell survival, tumor recurrence, and stem cell biology (18). All these processes are related to cancer drug resistance. Snail has been demonstrated to directly contribute to cisplatin resistance in breast cancer (19) and in ovarian cancer (20).

The Snail family of zinc-finger transcription factors comprises Snail (Snai1), Slug (Snai2), and Smuc (Snai3), all three of which share an evolutionarily conserved role in mesoderm formation in vertebrates (18). Structure of Snail family proteins includes a shared C-terminal domain, which is the most conserved feature and consists of four to six C_2_H_2_-type zinc fingers that mediate sequence-specific interactions with the DNA E-box (CAGGTG). DNA binding activity permits Snail to directly regulate E-cadherin expression and EMT progression (21).

However, the mechanisms of translational regulation of Snail by polarity protein remains poorly understood. In the present study, we demonstrate that reduction in Scribble protein levels serves as an initiation event conferring cell apoptosis and promoting cancer drug resistance through translocation of the RNA-binding protein HuR to the cytoplasm by activation of the p38 MAPK pathway, and permitting increased the EMT transcription factor *Snail* mRNA translation. These observations suggest a model for how loss of polarity proteins promotes drug resistance by regulating the EMT transcription factor Snail.

## Experimental Procedures

### 

#### 

##### Materials and Reagents

A549, CRL-1848, CRL-5803, and HTB-177 cells were maintained in RPMI 1640 (Hyclone, Logan, UT) medium. HEK293 and HeLa cells were maintained in DMEM (Hyclone). All media were supplemented with 10% fetal bovine serum (Hyclone) and 1% penicillin/streptomycin antibiotic stock solution (Gibco, Melbourne, Australia). All cell lines were obtained from the American Type Culture Collection (ATCC). For construction of Scribble KD and overexpression of the cell line, retrovirus was propagated in VSV-GPG cells and then transfected into the recipient, as previously described (9, 22). shRNA targeting HuR was subcloned into pLKO.1 and propagated in HEK293T cells, then transfected into the recipient. The resulting cell lines were maintained in basal cell culture medium as indicated above and supplemented with 4 μg/ml of puromycin (Sigma) along with cisplatin (Sigma) where indicated. Three-dimensional culture was performed as previously described (9). Actinomycin D, cycloheximide, protein degradation inhibitor MG132, p38 MAPK inhibitor SB203580, and isoproterenol were all purchased from Sigma.

##### Fluorescence-activated Cell Sorting (FACS) Analysis

Apoptotic cells were quantified using the Annexin V apoptosis detection kit (Bio-Vision, SF) according to the manufacturer's protocol. Cells were treated with cisplatin as indicated and analyzed using a FACS Canto II flow cytometer (BD Biosciences) and FlowJo software.

##### Cell Cycle Analysis

Cells were seeded at 5 × 10^5^ in a 6-cm plate and incubated overnight. The following day, cisplatin (Sigma) was added to the wells as indicated and incubated for 24 h. At analysis, cells were trypsinized, washed with PBS, and fixed overnight at 4 °C with 1 ml/well of 70% ethanol, followed by centrifugation at 2,000 × *g* for 5 min, washed with PBS, and incubated with 100 μg/ml of RNase (Sigma) at 37 °C for 15 min and staining with 50 μg/ml of propidium iodide (Sigma) for 15 min in the dark. The DNA content was analyzed in a Cell Lab Quanta SC flow cytometer (Beckman Coulter).

##### MTT Assay

Cells were seeded at 1 × 10^4^ cells/well in 96-well plates. The following day, different concentrations of cisplatin (Sigma) were added to the wells as indicated and incubated for 24 h. Following the addition 20 μl/well of MTT (thiazolyl blue tetrazolium bromide, 5 mg/ml, Sigma), plates were incubated for an additional 4 h. Culture medium was removed from the wells; dimethyl sulfoxide (Sigma) was added (100 μl/well) and plates were incubated for 20 min in a 37 °C incubator. Absorbance was measured with a microplate reader (Bio-Rad) at a wavelength of 590 nm. Each treatment was performed in four replicate wells. Each experiment was repeated three separate times.

##### Cell Lysis and Western Blotting

Cells were rinsed with ice-cold PBS and then lysed in RIPA buffer (Cell Signaling Technology) containing complete protease inhibitors (Roche Diagnostics) and phosphatase inhibitors (Roche Diagnostics), 5 mm dithiothreitol (DTT, Sigma), and 1 mm PMSF (Sigma) for 10 min on ice. Cells were then centrifuged at 15,000 × *g* for 10 min at 4 °C. Protein concentrations in the resulting supernatants were determined using the Bio-Rad protein assay (Bio-Rad) and samples were normalized for protein concentration. Aliquots containing 40 μg of total proteins each were loaded and separated by SDS-PAGE, then transferred to a PVDF membrane (Millipore) and stained by immunoblotting standard methods. The primary antibodies used for immunoblotting were anti-Scribble, anti-cleaved Caspase 3, anti-Slug, anti-Snail, anti-β-actin, anti-Hsp90, anti-PARP, anti-p38-MAPK, anti-phosphor-p38 MAPK, anti-eIF4E, anti-E-Cadherin, anti-N-Cadherin, anti-p-Akt (308), anti-Erk, anti-p-Erk, (1:1000; Cell Signaling Technology), anti-Twist, anti-HuR, and anti-Lamin B (1:1000, Santa Cruz).

##### Isolation of RNA, Reverse Transcription-PCR, and Quantitative Real Time-PCR

Total RNA was isolated using the Qiagen RNAeasy mini kit (Qiagen) according to the manufacturer's instructions. cDNA was generated by reverse transcription of 1-μg aliquots of RNA using the Takara PrimeScript RT Reagent Kit (Takara) according to the manufacturer's protocol. The cDNA was used for real time-PCR using the SYBR Premix Ex-Taq Kit (Takara) on a CFX96 instrument real time PCR system (Bio-Rad). All expression data were normalized to β-actin-encoding transcript levels. Primers sequences were as follows: Scribble-F, 5′-GGGACGACGAGGGCATATTC-3′, Scribble-R, 5′-CGTTCTCAGGCTCCACCATGC-3′; Snail-F, 5′-GCTGCAGGACTCTAATCCAGA-3′, Snail-R, 5′-ATCTCCGGAGGTGGATG-3′; Slug-F, 5′-CCAAACTACAGCGAACTGGA-3′, Slug-R, 5′-GTGGTATGACAGGCATGGAG-3′; Twist-F, 5′-GGAGTCCGCAGTCTTACGAG-3′, Twist-R, 5′-TCTGGAGGACCTGGTAGAGG-3′; β-actin-F, 5′-CCTGCACCCACACAAT-3′, β-actin-R, 5′-GGGCGGACTCGTCAAC-3′; 18S-F 5′-CAGCCACCCGAGATTGAGCA-3′, 18S-R 5′-TAGTAGCGACGGGCGGTGTG-3′.

##### siRNA Transfections

*Scribble* siRNA oligonucleotides and *HuR* siRNA oligonucleotides were purchased from Santa Cruz Biotechnology, Inc. The siRNA was transfected into CRL-1848 cells using FuGENE HD Transfection reagent (Promega) according to the manufacturer's instructions.

##### Immunofluorescence and Confocal Microscopy

Cells plated on coverslips were rinsed twice with PBS and fixed with 4% paraformaldehyde for 30 min at room temperature. Cells then were washed 3 times with PBS, permeabilized (10 min) in 0.4% Triton X-100 for 10 min, and blocked (1 h at room temperature) in PBS with 0.5% Tween 20 (PBST) containing 4% bovine serum albumin (Sigma). After overnight incubation at 4 °C with the indicated primary antibody, cells were washed with PBS (3 × 10 min) and then incubated (2 h at room temperature) with Alexa Fluro 568 goat anti-rabbit IgG (Invitrogen) or Alexa 568 goat anti-mouse IgG (Invitrogen). After washing with PBS (3 × 10 min), cells were exposed for 5 min to 0.5 μg/ml of DAPI (Sigma). Following final washes with PBS (3 × 10 min), the coverslips were mounted using Fluoromount Aqueous Mounting Medium (Sigma) and imaged using an Olympus Fluoview FV1000 confocal laser scanning microscope. Raw images were analyzed using the Olympus FV10-ASW 2.1 Viewer software (Olympus).

##### Polyribosome Gradient Analyses

Polyribosome profile analyses were performed as previously described with minor modifications (23). 5 × 10^6^ cells were cultured in medium with 0.1 mg/ml of cycloheximide (Sigma) at 37 °C for 10 min, and then washed twice with cold PBS containing 0.1 mg/ml of cycloheximide. Cells were harvested and lysed by incubation on ice for 10 min in 1 ml of PEB lysis buffer (20 mm Tris-HCl, pH 7.5, 100 mm KCl, 5 mm MgCl_2_, 0.3% (v/v) Triton X-100, 0.1 mg/ml of cycloheximide, 1 mm DTT, 200 units/ml of RNase out (Takara), 1× complete protease inhibitor mixture (Roche Diagnostics)). Lysed cells were centrifuged at 13,000 × *g* for 10 min at 4 °C. Protein concentration of the cytoplasmic lysate was measured as above. Aliquots containing ∼2.5 mg of cytoplasmic lysate protein were layered on top of linear 10–50% (w/v) sucrose gradients. Tubes were centrifuged in a Beckman SW41 rotor (Beckman) at 260,500 × *g* for 2 h at 4 °C. The entire centrifuged content was divided into 12 fractions from top to bottom as polyribosome profiles. Fine fractions of those 12 fractions are acquired from the gradients and that can be measured by qualitative RT-PCR and Western blot assay. RNA in 300 μl of each fraction was extracted by using TRIzol/chloroform/isopropyl alcohol and reverse transcribed as described above. RNA samples were assayed for levels of *Snail* transcript and 18S rRNA by real-time PCR as described above. To measure the RNA-binding protein abundances in various fractions, we used 100 μl of each fraction for Western blot analyses as described above.

##### Nuclear and Cytoplasmic Protein Extraction

Nuclear and cytoplasmic proteins were extracted using NE-PER nuclear and cytopalsmic extraction reagents (Thermo Scientific) according to the manufacturer's protocol. Briefly, cells were harvested by adding typsin-EDTA washed with ice-cold PBS and then lysed in ice-cold CER I for 15 min, followed by addition of ice-cold CER II and incubation on ice for 1 min. Cells then were centrifuged at 16,000 × *g* for 5 min and the supernatant containing the cytoplasmic proteins was removed and stored for use. The insoluble pellet was resuspended in ice-cold NER and incubated on ice for 40 min, centrifuged the tube at maximum speed for 10 min, immediately transferred to the supernatant fraction to a clean tube, and extracts were stored at −20 °C until use.

##### Xenograft Experiments

All animal experiments were performed in accordance with a protocol approved by the Institutional Animal Care and Use Committee of the Institute for Nutritional Sciences, Shanghai Institutes for Biologic Sciences, and Chinese Academy of Sciences. On day 0, nude mice (4 to 8 weeks old) were injected subcutaneously on the right flank with the following tumor cells (1 × 10^6^/mouse) in 25 μl of serum-free medium (mixed with Matrigel at a 1:1 ratio). Tumor growth was monitored every other day. Tumor volume was calculated as (width 2 × length/2). When tumors became established at the desired size (about 1 week after tumor cell implantation), mice harboring palpable tumors were weighed and randomly assorted into control and treatment groups (10 mice each). Cisplatin was dissolved in physiological saline and administered (via intraperitoneal injection; every other day for 15 days) at doses of 2.5 or 5 mg/kg. The control group received equivalent volumes of saline only. The body weights were recorded 1 day after the final cisplatin dose.

##### Statistics

Data were depicted as mean ± S.D. from at least three independent experiments. Two-tailed Student's *t* tests were performed using Instat 5.0 (GraphPad). Exact *p* values are provided in each figure. *p* values of <0.05 were considered significant.

## Results

### 

#### 

##### Loss of Scribble Enhances Cisplatin-related Drug Resistance

**FIGURE 1.**
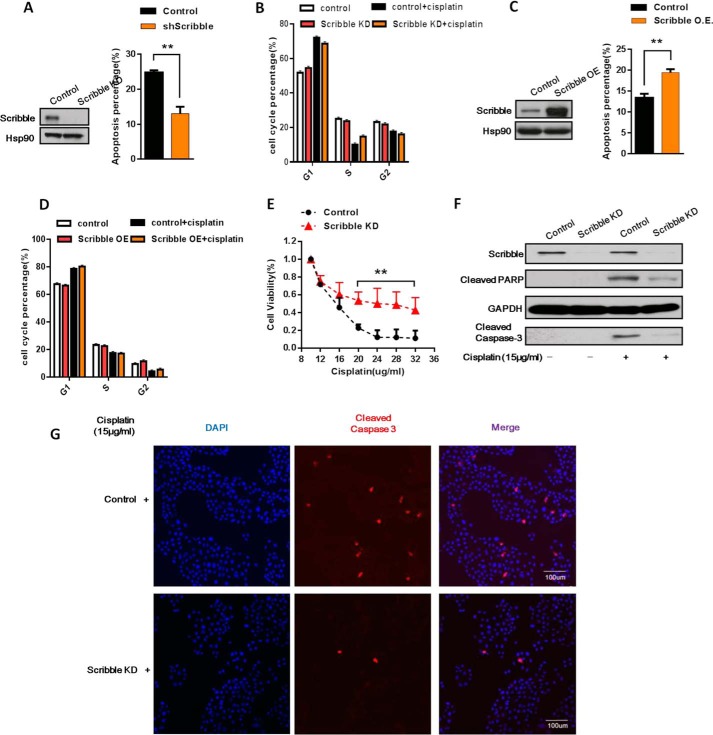
**Knockdown of *Scribble* promotes cisplatin-related drug resistance.** *A,* apoptotic cells were quantified in Scribble knockdown (*KD*) cells in the CRL-1848 cell line following cisplatin treatment (15 μg/ml, 24 h). Apoptosis was measured using an annexin V apoptosis detection kit and expressed as the percentage of total cells that were in late apoptosis or were both annexin V-PE and PI positive. *Left side* was the Scribble KD efficiency examined by Western blot. *B,* cell cycle distributions was measured using flow-cytometric analysis in Scribble KD cells in CRL-1848 cell line when compared with control cells under cisplatin treatment. *C,* apoptosis was measured using annexin V detection kit in Scribble overexpression (*O.E*.) cells in the HTB-177 cell line, *left side* was the Scribble overexpression efficiency examined by Western blot. *D,* cell cycle distributions were measured using flow-cytometric analysis in Scribble overexpression HTB-177 cells when compared with control cells under cisplatin treatment. *E,* the CRL-1848 cell line Scribble KD or control cells were treated with different concentrations of cisplatin for 24 h. Cell viability was determined using an MTT assay; cell viability was expressed as the percent of total cell number. *F,* immunoblots of untreated and 24-h cisplatin-treated cells (15 μg/ml, 24 h) in the CRL-1848 cell line control and Scribble KD cells. Primary antibodies were specific for Scribble, cleaved PARP, cleaved caspase 3 or GAPDH (loading control). *G*, immunofluorescence of Scribble KD or control cells following 24 h of cisplatin exposure in the CRL-1848 cell line. *Left column* (*blue*), DAPI (nuclei); *middle column* (*red*), cleaved caspase 3; *right column,* merged images.

To test the effects of the polarity gene *Scribble* on the cellular process of apoptosis, we generated stable *Scribble* KD gene expression in different cancer cell lines. We observed that there was no significant difference in the percentage of apoptosis between Scribble KD cells and control cells by general culture conditions. The platinum chemotherapeutic agent cisplatin is the first-line for lung cancer patients (24). Surprisingly, following cisplatin treatment, Scribble KD lines exhibited significant attenuation in the percentage of apoptotic cells compared with control cells (Fig. 1*A*). Meanwhile, flow-cytometric analysis documented that G_1_ phase, S phase, and G_2_/M phase remained almost the same in control cells, Scribble KD cells, and Scribble overexpression cells. Consistent with previous data in mammary gland cells (9), it indicated that Scribble low expression had no significant effect on the regulation of cell cycle and proliferative activity (Fig. 1*B*). Moreover, cells engineered to overexpress *Scribble* exhibited significantly increased levels of apoptosis upon cisplatin treatment (Fig. 1*C*). Meanwhile, there is no significant difference in cell cycle distributions in Scribble overexpression HTB-177 cells when compared with control cells under cisplatin treatment (Fig. 1*D*). To determine whether Scribble KD cells developed resistance to cisplatin, cells were treated with different concentrations of cisplatin for 24 h. A cell viability assay revealed that the percentage of surviving cells was enhanced by Scribble KD (Fig. 1*E*). The 50% inhibition concentration (IC_50_) for cisplatin in control and Scribble KD cells was 16.28 ± 0.06 and 26.33 ± 6.08 μg/ml, respectively. These data suggested that loss of Scribble can enhance cellular drug resistance to cisplatin.

Caspase 3 has been identified as a key mediator of apoptosis and it orchestrates the demolition of cell death by cleaving several key proteins required for cellular function and survival. For instance, poly(ADP-ribose) polymerase (PARP) can be cleavaged by caspase 3 to the inactivate form, rendering the cell unable to respond to breaks in DNA strands (25, 26). Western blot assays showed that cleaved caspase 3 and cleaved PARP levels were decreased in Scribble KD cells compared with control cells in cisplatin treatment (Fig. 1*F*). Consistent with this notion, immunofluorescence studies in cisplatin-exposed cells also revealed decreased staining for cleaved caspase 3 in Scribble KD cells (Fig. 1*G*). These results showed that apoptosis was decreased by KD of Scribble, suggesting that loss of Scribble promoted drug resistance to cisplatin by suppressing apoptosis.

##### Scribble Can Regulate Snail to Mediate Drug Resistance

**FIGURE 2.**
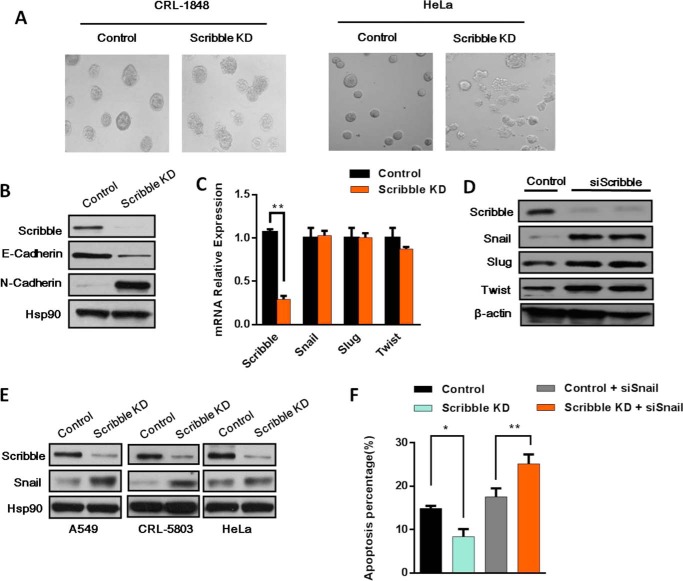
**Scribble regulation of drug resistance is mediated by Snail.** *A,* phase images of day 12 control and Scribble KD cells in the CRL-1848 and HeLa cell lines in three-dimensional cell culture (×200 magnified). *B,* EMT markers were examined in Scribble KD cells or control cells in the CRL-1848 cell line by Western blot. *C,* EMT transcription factors Snail, Slug, and Twist mRNA levels were examined in CRL-1848 Scribble KD cells and control cells by RT-PCR. *D,* two different siRNAs were used to inhibit *Scribble* expression in the CRL-1848 cell line. The accumulation of EMT transcription factors Snail, Slug, and Twist were examined by Western blotting. β-Actin was used as a loading control. *E,* the protein levels of Scribble, Snail, and Hsp90 (loading control) were examined by Western blotting in three different Scribble KD cancer cell lines (A549, CRL-5803, and HeLa). *F,* apoptosis was measured in cisplatin-treated (15 μg/ml, 24 h) cells using the annexin V apoptosis assay. Cells were transfected with constructs for control; Scribble KD; control + siSnail; or Scribble KD + siSnail.

To investigate the cellular effects of Scribble in epithelial cells, we established cell lines stably expressing Scribble shRNA and developed a three-dimensional culture model (22). Surprisingly, when propagated in this three-dimensional culture system, Scribble KD cells exhibited an enhanced ability to invade through Matrigel-impregnated membranes, and disrupted three-dimensional acinar structures generated in a non-small lung cancer cell (NSLCC) line (CRL-1848) and HeLa cells (Fig. 2*A*). However, Scribble KD lines did not exhibit apparent morphological differences compared with control cells when grown in two-dimensional cultures (data not shown). Despite an apparent lack of morphological effect, Scribble KD lines showed changes in E-cadherin and N-cadherin protein levels in a two-dimensional culture (Fig. 2*B*), with Scribble KD lines exhibiting a mesenchymal phenotype for production of these two cadherins production, a signature that serves as a marker of EMT fate.

Given our observations that Scribble KD promoted drug resistance to cisplatin, we wondered how Scribble regulated drug resistance. We speculated that Scribble KD may promote drug resistance by directly targeting an EMT transcription factor. To investigate this hypothesis, we examined the mRNA level of several EMT transcriptional factors (Twist, Snail, and Slug) in Scribble KD and control cells. But there was no significant change among these factors (Fig. 2*C*). Next, CRL-1848 cells were transfected with a synthetic siRNA targeting *Scribble* or with a control siRNA. To exclude off-target effects, two different *Scribble* siRNAs were used. Scribble KD efficiency was detected by Western blotting at 48 h after transfection. As shown in Fig. 2*D*, Scribble protein levels were decreased greater than 10-fold following transfection with either of the *Scribble* siRNAs. We additionally assessed the protein levels of the EMT transcription factors (Twist, Snail, and Slug), and observed a marked accumulation of Snail but slight accumulation of Slug and Twist (Fig. 2*A*). To confirm that these effects were not cell line-specific, we performed Scribble KD in 3 other cell lines. Each line yielded similar results, with levels of Snail increased upon Scribble KD compared with the respective control line (Fig. 2*E*). These results suggested that Scribble can regulate Snail accumulation, in a reverse of the “classic” regulation of polarity proteins by EMT transcription factors.

Our observations suggested that the role of Scribble in cisplatin resistance may be mediated by regulation of Snail expression. To test this hypothesis, we analyzed cells using an annexin V apoptosis detection kit and siRNA-mediated inhibition of *Snail* expression. This analysis revealed that cells with Snail KD exhibited increased apoptosis. Notably, KD of Snail in Scribble KD cells restored apoptosis in response to cisplatin treatment (Fig. 2*F*). Together, these observations suggested that the KD of Scribble resulted in increased Snail accumulation, and that effects on Snail were the primary mechanism of increased drug resistance.

##### Scribble KD Redistributes Snail mRNA in Polyribosomes and Increases Translation of the Snail Transcript

**FIGURE 3.**
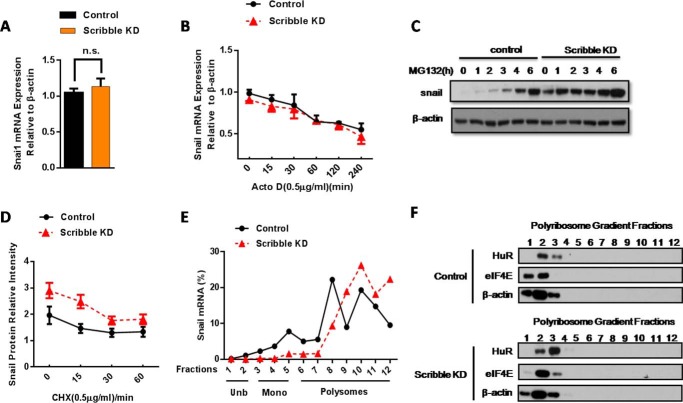
**Scribble KD alters polyribosome distribution of *Snail* mRNA and increases translation of the *Snail* mRNA.** *A,* Snail mRNA levels were not significantly changed in Scribble KD cells, as assessed by RT-PCR. *B, Snail* mRNA stability did not differ significantly between Scribble KD cells and control cells during up to 4 h of exposure to the transcription inhibitor actinomycin D (0.5 μg/ml). *C,* Snail protein synthesis rates were measured in a time course by proteasome inhibitor MG132 (20 μm) in Scribble KD and control cells. *D,* Snail protein degradation rates (indicated by slope of lines) did not differ significantly between Scribble KD and control cells following exposure to translation inhibitor cycloheximide (*CHX*, 0.5 μg/ml). *E,* relative polyribosome distribution of *Snail* mRNA in Scribble KD and control cells was analyzed by sucrose gradient centrifugation. *mRNPs*, messenger ribonucleoprotein particles. *Unb*, unbound RNPs, Fractions 1 and 2. *Mono*, monoRNPs (40S, 60S, 80S), fractions 3–5. Polysomes: polyribosomes, fractions 6–11. Representative data are from three independent experiments. *F,* analysis of RNP protein content in polyribosome fractions. Polyribosome distributions (12 fractions each) from control and Scribble KD cells in the CRL-1848 cell line were isolated as above, and RNA-binding protein content was analyzed by Western blotting; β-actin served as an internal control.

To analyze the molecular mechanism of the effect of Scribble on Snail accumulation, we examined *Snail* mRNA levels and *Snail* mRNA stability upon exposure to the transcription inhibitor actinomycin D. Neither the level nor stability of the transcript were significantly changed in response to Scribble KD compared with control cells (Fig. 3, *A* and *B*), suggesting that Scribble may regulate Snail accumulation at a post-transcriptional level. Following administration of MG132 at various time points, the rate of Snail protein synthesis was higher in Scribble KD cells while compared with control cells (Fig. 3*C*). The results from the time course of treatment in Scribble KD cells with cycloheximide, a protein synthesis inhibitor, indicated no apparent changes in the degradation or the half-life of Snail protein (Fig. 3*D*). We therefore supposed that Scribble regulates Snail accumulation at the level of translation. To further substantiate this impact of Scribble KD on Snail translation, we performed polyribosome profiling of cytoplasmic lysates from Scribble KD and control cells. In control cells, ∼25% of *Snail* mRNA co-sedimented with unbound ribonucleoprotein particles (RNPs) and mono-bound RNPs, and the remaining ∼75% of *Snail* mRNA co-sedimented with polyribosomes. In KD Scribble cells, approximately ∼90% of *Snail* mRNA co-sedimented with polyribosomes (Fig. 3*E*). These results indicated that the KD of Scribble increased the proportion of *Snail* mRNA in polyribosome fractions, yielding increased translation of *Snail* transcript.

We next sought to define the RNA-binding proteins involved in *Snail* mRNA translation. eIF4E, which belong to the eukaryotic initiation factors (eIFs) family and control the initiation step of translation, is present at lower abundance than other translation factors, consistent with its being a rate-limiting factor in translation (27). Adenylate- and uridylate-rich elements (AREs) are increasingly appreciated as central elements of gene regulation. More recently, AREs have also been reported to participate in translation. HuR binds with high affinity and specificity to target mRNAs through ARE recognition motifs, thereby facilitating translation (11, 28). Interestingly, according to Dong *et al.* (29), there are 3 AREs in the 3′-UTR of the *Snail* mRNA that can be recognized by HuR and mediate Snail mRNA stability. We analyzed the relative concentrations of HuR and eIF4E proteins in different fractions of polyribosome profiles. Notably, Scribble KD stimulated a shift in the distribution of HuR and eIF4E proteins into mono-bound RNPs (Fig. 3*F*). As an internal control, we demonstrated that there was no change in β-actin-encoding mRNA distribution between Scribble KD and control cells (Fig. 3*F*). Taken together, these results indicate that loss of Scribble yielded increased RNA-binding protein binding to *Snail* mRNA and elevated translation of the Snail transcript. Thus, these observations strengthen our notion that Scribble KD increases *Snail* mRNA translation and promotes drug resistance.

##### HuR KD Increased Cisplatin Sensitivity and Apoptosis

**FIGURE 4.**
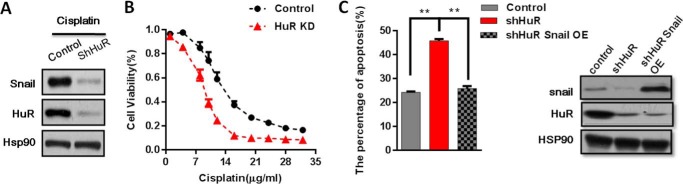
**HuR KD cells increased apoptosis and sensitivity to cisplatin.** *A,* KD HuR decreased the Snail protein level in cisplatin-treated (15 μg/ml, 24 h) cells. Hsp90 served as a loading control. *B,* the CRL-1848 cell line HuR KD or control cells were treated with different concentrations of cisplatin for 24 h. Cell viability was determined using an MTT assay; cell viability was expressed as the percent of total cell number. *C,* Western blot analysis of HuR KD and Snail expression in the CRL-1848 cell line, *left side,* apoptotic cells were quantified in HuR KD and snail overexpression in the CRL-1848 cell line following cisplatin treatment (15 μg/ml, 24 h). The annexin V apoptosis detection kit for the quantification of apoptotic cells was used.

To assess the role of HuR in drug resistance to cisplatin. We constructed a stable HuR KD cell line. We observed that KD HuR resulted in reduced Snail protein levels (Fig. 4*A*). Additionally, Snail accumulation was also decreased in cisplatin-treated HuR KD cells (Fig. 4*A*). To determine the role of HuR in cisplatin-related resistance to cisplatin, we performed a cell viability assay. We found that HuR KD cells had restored sensitivity to cisplatin when CRL-1848 HuR KD cells were treated with different concentrations of cisplatin for 24 h. The 50% inhibition concentration (IC_50_) for cisplatin in control and HuR KD cells was 14.09 ± 0.81 and 6.05 ± 0.37 μg/ml, respectively. It revealed that the percentage of surviving cells was reduced by HuR KD. We demonstrated that HuR KD can increase cisplatin-induced apoptosis by using an annexin V apoptosis detection kit (Fig. 4*C*). Ectopic overexpression of Snail in HuR KD cells partially attenuated HuR KD increased apoptosis during cisplatin treatment (Fig. 4*C*). Taken together, these results revealed that HuR KD can increase cisplatin-induced apoptosis through down-regulation of the Snail protein.

##### HuR Can Be Activated and Translocated to the Cytoplasm upon Scribble KD

**FIGURE 5.**
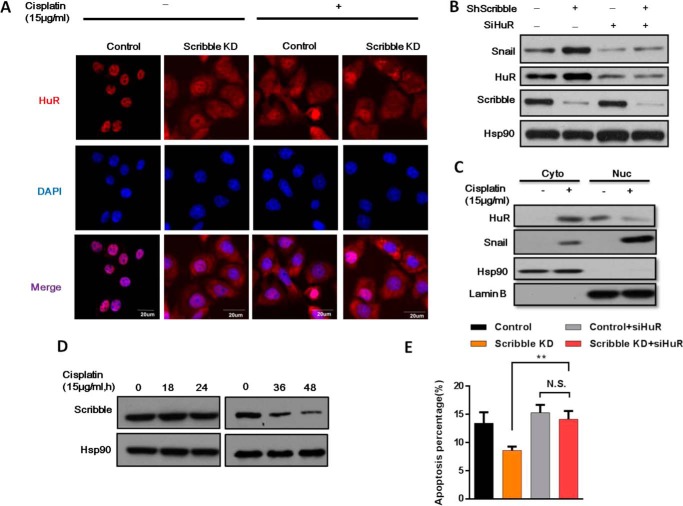
**HuR is translocated to the cytoplasm by Scribble KD.** *A,* Scribble KD induced translocation of HuR from the nucleus to cytoplasm. Control and Scribble KD cells were treated with or without cisplatin (15 μg/ml) for 12 h, and HuR (*red*) and nuclear (DAPI; *blue*) were detected by immunofluorescence staining. *B,* Snail accumulation was reduced upon silencing of HuR in Scribble KD cells. Control and Scribble KD cells were subjected to HuR silencing, and protein levels were detected by Western blotting. *C,* the unclear and cytoplasmic localization of HuR along with cisplatin treatment was detected by Western blot. *D,* the Scribble expression level was measured in a time course after the short term (≤24 h) or long term (2 days) exposure to the cisplatin by Western blot. *E,* apoptosis was measured in cisplatin-treated (15 μg/ml, 24 h) cells using the annexin V apoptosis assay. Cells were transfected with constructs for control, Scribble KD, control + siHuR, or Scribble KD + siHuR.

It has been reported that HuR, a protein located primarily in the nucleus, can shuttle between the nucleus and cytoplasm in a process that depends on the nuclear-cytoplasmic shuttling sequence (30). Given that translation is a cytoplasmic process, we hypothesized that Scribble KD activates translocation of HuR to the cytoplasm, thereby promoting HuR-mediated translation of the Snail transcript. Therefore, immunofluorescence staining demonstrated that HuR was predominantly localized in the nucleus in control cells, whereas cytoplasmic HuR levels were increased in Scribble KD cells (Fig. 5*A*). To assess whether Scribble KD influenced Snail accumulation through HuR, we investigated the specific effect of Scribble KD on HuR activity. As expected, the expression of HuR was lightly increased in Scribble KD cells (Fig. 5*B*). Furthermore, HuR was translocated to the cytoplasm exposure by cisplatin both in control cells and Scribble KD cells (Fig. 5, *A* and *C*). Indeed, Snail protein accumulation in Scribble KD cells was further reduced by silencing HuR in Scribble KD cells during cisplatin treatment (Fig. 5*B*). Notably, we tested the expression level of Scribble in a time course after short (≤24 h) or long term (36 and 48 h) exposure to cisplatin. There was no change in the level of Scribble expression upon short term cisplatin treatment, meanwhile there was a slightly attenuated Scribble protein level in response to long term cisplatin treatment. It indicated that Scribble low expression cells may be related to drug resistance after long exposure of cisplatin treatment. We analyzed cells using an annexin V apoptosis detection kit and siRNA-mediated inhibition of *HuR* expression. It revealed that cells with HuR KD in the Scribble KD cell significantly increased apoptosis compared with Scribble KD cells in response to cisplatin treatment (Fig. 5*E*). Collectively, these data demonstrated that HuR was relocated to the cytoplasm in Scribble KD cells, providing a mechanism for elevated *Snail* mRNA translation and increased drug resistance.

##### Loss of Scribble Regulates Apoptosis and Snail mRNA Polyribosomes Distribution Depend on p38 MAPK Pathway

**FIGURE 6.**
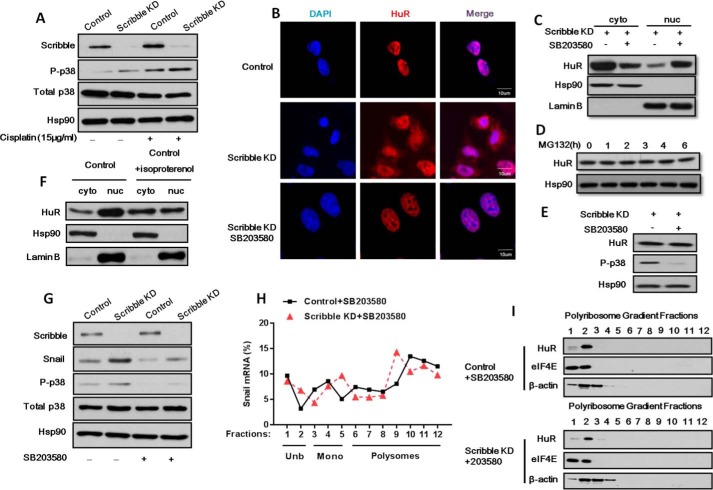
**Loss of Scribble mediates drug resistance by activation of p38 MAPK pathway.** *A,* phosphorylated p38 MAPK (p-p38) was activated in Scribble KD cells. *B,* the p38 MAPK inhibitor prevented Scribble KD-mediated HuR cytoplasmic translocation. Control and Scribble KD cells were treated with or without p38 MAPK inhibitor (SB203580; 10 μm, 12 h), and HuR (*red*) and nuclear (DAPI; *blue*) were detected by immunofluorescence staining. *C,* the translocation of HuR to the cytoplasm in Scribble KD cells was counteracted by exposure to the p38 MAPK inhibitor (SB203580, 10 μm, 12 h). Scribble KD cells were separated into cytosolic (Cyto) and nuclear (*nuc*) fractions, HuR, Lamin B (nuclear fraction control; loading control), and Hsp90 (cytosolic fraction control; loading control) were detected by Western blotting. *D,* HuR protein levels were measured in a time course by the proteasome inhibitor MG132 (20 μm) in Scribble KD and control cells. *E,* HuR protein levels were not significantly changed when Scribble KD cells were treated with SB203580, as assessed by Western blot. *F*, cytosolic and nuclear distribution detected in control cells were treated with or without p38 MAPK agonist (isoproterenol; 10 μm, 12 h) and processed as in *C. G,* Snail accumulation in CRL-1848 Scribble KD cells was decreased by SB203580 treatment (10 μm, 12 h). *H*, relative polyribosome distribution of *Snail* mRNA in the CRL-1848 cell line control and Scribble KD cells by SB203580 treatment (10 μm, 12 h) analyzed by sucrose gradient centrifugation as indicated above. *I,* analysis of RNP protein content in polyribosome fractions. Polyribosome distributions (12 fractions each) from the CRL-1848 cell line control and Scribble KD cells by SB203580 treatment (10 μm, 12 h) were isolated as above, and RNA-binding protein content was analyzed by Western blotting; β-actin served as an internal control.

Loss of Scribble-mediated HuR in drug resistance has not previously been reported, to our knowledge. We analyzed several candidate signaling pathways for potential contributions to this process, and found that p-p38 MAPK was increased in Scribble KD cells (Fig. 6*A*). To determine whether the p38 MAPK signaling pathway was involved in Scribble KD-mediated HuR translocation, we treated the cells with selective inhibitors and performed immunofluorescent staining using an antibody against HuR. We observed that SB203580, an inhibitor of p38 MAPK, blocked HuR nuclear export (Fig. 6*B*). To further investigate this finding, we assessed subcellular distribution of the HuR protein in Scribble KD cells treated with SB203580. We observed that translocation of HuR to the cytoplasm in Scribble KD cells was counteracted by exposure to the p38 MAPK inhibitor (Fig. 6*C*). As shown in Fig. 6*D*, MG132 treatment had no effect on HuR degradation. We observed no change of the total HuR protein level when Scribble KD cells were treated with SB (Fig. 6*E*). It indicated that p38-induced cytoplasmic accumulation was not due to HuR degradation. Consistent with the proposed role of the p38 MAPK pathway, treatment of control cells with isoproterenol, a p38 agonist, also increased HuR translocation to the cytoplasm (Fig. 6*F*). We further assessed whether activation of p38 MAPK can mediate *Snail* accumulation and transcript translation in Scribble KD cells. We examined the Snail protein expression level after treatment of SB203580. As expected, the Snail level was decreased following p38 MAPK inhibitor treatment in Scribble KD cells (Fig. 6*G*). We then performed polyribosome profiling of cytoplasmic lysates in Scribble KD and control cells following treatment of SB203580. The increased proportion of *Snail* mRNA in polyribosome fractions in Scribble KD cells without SB203580 disappeared when compared with control inhibition of p38 MAPK with SB203580 (Fig. 6*H*). Consistent with this finding, we analyzed the relative concentrations of HuR and eIF4E proteins in different fractions of polyribosome profiles. Notably, there was no shift in distribution of HuR and eIF4E proteins into RNPs between control and Scribble KD cells when treated with SB203580, as well as an internal control β-actin-encoding mRNA distribution (Fig. 6*I*). Taken together, these results indicate that loss of Scribble yielded increased RNA-binding protein binding to *Snail* mRNA and elevated translation of the Snail transcript depends on p38 MAPK activity. Collectively, these data indicated that the loss of Scribble activated the p38 MAPK signaling pathway, contributing to HuR translocation, resulting in increased *Snail* mRNA translation and promoting drug resistance.

##### Loss of Scribble Induces Cisplatin Resistance Both in Vitro and in Vivo

**FIGURE 7.**
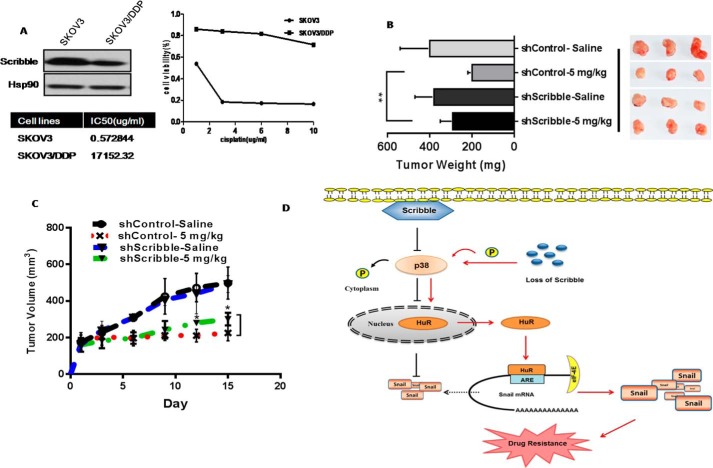
**Loss of Scribble mediates drug resistance both *in vivo* and *in vitro*.** *A,* protein levels of Scribble in the indicated cisplatin-resistant SKOV3/DDP cell lines (and respective parent lines) were detected by immunoblotting with anti-Scrib and HSP90. HSP90 served as a loading control, the MTT assay was used to assess the relative survival of cells harboring the control RNAi or Scribble RNAi after treatment with cisplatin at the indicated concentrations. Assays were performed in triplicate and the results are presented as the mean ± S.D. *B,* nude mice harboring tumors derived from implanted control A549 or A549 Scribble KD cells were treated by intraperitoneal injection with saline or 2.5 mg/kg of cisplatin. Tumor weights calculated are presented as the mean ± S.D. for each group (*n* = 4), and their weight plotted in comparison to mice. **, *p* < 0.01 (Student's *t* test). *C,* tumor dimensions were recorded; *, *p* < 0.05 (Student's *t* test). *D,* a model proposed mechanism for Scribble regulation of p38 MAPK activity and HuR translocation, resulting in increased Snail translation and promotion of drug resistance. *P* in *circles* indicates phosphorylation.

We have developed panels of a cisplatin-resistant cancer cell line, SKOV3/DDP, with acquired and inherent resistance to cisplatin. We further demonstrated that the SKOV3/DDP cell line had a lower Scribble protein level when compared with the parental cells. To directly test the *in vivo* Scribble function in cisplatin sensitivity, we performed xenografts experiments with control or the stable KD human lung cancer cell line A549 cells following cisplatin treatment. As shown in Fig. 7, *B* and *C*, among the cisplatin-treated animals, tumors in mice implanted with A549 Scribble KD cells remained significantly larger and grew faster. The present study suggests the importance of Scribble in cisplatin resistance.

## Discussion

These findings provide a novel insight into how Scribble, a polarity protein, plays a role in the regulation of the RNA-binding protein HuR and works as a novel regulator of EMT transcription factor Snail. Additionally, we observed an EMT phenotype in many Scribble KD human cancer cell lines using a three-dimensional cell culture system. Although we saw no typical EMT phenotype in the two-dimensional culture system, but consistently observed that loss of Scribble resulted in Snail accumulation in cancer cells.

EMT has been observed in tumor samples from patients with non-small cell lung cancer (NSCLC) that developed resistance to epidermal growth factor receptor inhibitors (31, 32), and with pancreatic cancer that developed resistance to gemcitabine (33). The role of EMT in drug resistance likely relies on many of the same transcription factors (Snail, Slug, and Twist) that function in cancer metastasis, but the mechanism of regulation of these factors remains largely unknown. It has been shown that there are binding sites for several EMT transcription factors in several ATP-binding cassette transporters associated with development of multidrug resistance (34). Thus, accumulating evidence supports the idea that EMT transcription factors play a distinct role in drug resistance. Our work supports the idea that deregulation of Scribble may play a potential role in drug resistance by a direct impact on accumulation of the EMT transcription factor Snail.

Previous work uncovered that Snail was involved in the acquisition of stem cell-like characteristics (35). Ectopic expression of Snail greatly increased the tumor cells' ability to form mammospheres, the formation of which is a signature property of mammary epithelial stem cells (18). It was recently demonstrated that Scribble interacted with Lats2 and TAZ to mediate the Hippo pathway and regulate cancer stem cell population (36, 37). Lats2 can directly phosphorylate Snail, resulting in retention of Snail within the nucleus and thereby enhancing its stability (17). These reports suggest that Scribble and Snail have a cross-link in regulation of cancer stem cell development. But the role of the polarity protein Scribble to mediate Snail levels in apoptosis and drug resistance is not well known. Based on our findings, we hypothesize that Scribble regulates p38 MAPK activity to balance HuR subcellular distribution, thereby controlling Snail translation. We demonstrated that deregulation of Scribble enhances accumulation of the Snail protein in cancer cells, and that effects on Snail were the primary mechanism of cisplatin-related drug resistance. Further dissection of the molecular aspects of regulation of Snail translation promises to identify additional molecular targets that could provide new therapeutic strategies for overcoming drug resistance.

Finally, our studies reveal a significant function for Scribble in regulating the p38 MAPK signaling pathway. We show that KD of Scribble in cancer cell lines results in drug resistance and inhibition of apoptosis, effects that are mediated through activation of the p38 MAPK pathway. There is evidence that Scribble can directly regulate Akt through formation of a trimetric complex with PHLPP (38). ERK also interacts with Scribble through two conserved kinase interaction motif docking sites (39). It has been reported that Scribble can act as a scaffold protein, connecting Mst1 and PHLPP (38, 40), and permitting PHLPP dephosphorylating of Mst1, which in turn activates the downstream effector p38 MAPK. Our data suggests that Scribble can mediate cancer cell apoptosis by an unexpected functional regulation of RNA-binding protein HuR, in a MAPK family member p38 activated manner. Our findings, taken together with those of others, provide an important extension to our understanding of the role of Scribble in controlling MAPK signaling pathways.

In summary, we demonstrated that Scribble KD promoted drug resistance by inhibiting cancer cell apoptosis following cisplatin treatment, and that this inhibition was related to increased accumulation of Snail in Scribble KD cells. Further experiments revealed that p38 MAPK was activated in Scribble KD cells, thereby promoting HuR translocation into the cytoplasm and facilitating increased accumulation of Snail protein by increasing its synthesis (Fig. 7). These findings highlight how a polarity protein regulates EMT transcription factor Snail, and how deregulation of polarity protein Scribble mediates cancer drug resistance.

## Author Contributions

L. Z. designed the research; Y. Z., R. C., W. J., J. G., N. W., Y. X., and M. Q. performed the research; Y. Z., R. C., and L. Z. analyzed the data; Y. Z. and L. Z. wrote the manuscript. All authors reviewed the results and approved the final version of the manuscript.

## Footnotes

3 The abbreviations used are:

HuR: human antigen R
PARP: poly(ADP-ribose) polymerase
KD: knockdown
MTT: 3-(4,5-dimethylthiazol-2-yl)-2,5-diphenyltetrazolium bromide
RNP: ribonucleoprotein particle
ARE: AU-rich element
EMT: epithelial-mesenchymal transition.
